# Low circadian clock genes expression in cancers: A meta-analysis of its association with clinicopathological features and prognosis

**DOI:** 10.1371/journal.pone.0233508

**Published:** 2020-05-21

**Authors:** Jiangguo Zhang, Hong Lv, Mingzhu Ji, Zhimo Wang, Wenqing Wu

**Affiliations:** 1 Department of Gastroenterology, Shekou People’s Hospital, Shenzhen, Guangdong, China; 2 Department of Gastroenterology, Nanshan People’s Hospital, Shenzhen, Guangdong, China; 3 Shekou People’s Hospital, Shenzhen, Guangdong, China; Texas A&M University, UNITED STATES

## Abstract

**Background:**

Per1, Per2, Per3, Cry1, Cry2, Bmal1, Npas2 and CLOCK genes are the eight core circadian clock genes. Low expression of these circadian clock genes plays an important role in the progression of cancers. However, its clinicopathological and prognostic value in patients with cancers remains controversial and inconclusive. We performed a meta-analysis of studies assessing the clinicopathological and prognostic significance of low expression of these genes in cancers.

**Methods:**

Relevant studies were searched from the Cochrane Central Register of Controlled Trials, Embase, EBSCO, Ovid, PubMed, Science Direct, Wiley Online Library database, CNKI and Wan Fang database. The meta-analysis was performed by using STATA version 12 software. A random-effect model was employed to evaluate all pooled hazard ratios (HRs) and odd ratios (ORs).

**Results:**

A total of 36 studies comprising 7476 cases met the inclusion criteria. Meta-analysis suggested that low expression of Per1 was associated with poor differentiation (Per1: OR=2.30, 95%CI: 1.36∼3.87, *P*=0.002) and deeper invasion depth (Per1: OR=2.12, 95%CI: 1.62∼2.77, *Ρ*<0.001); low Per2 expression was correlated with poor differentiation (Per2: OR=2.41, 95%CI: 1.53∼3.79, *Ρ*<0.001), worse TNM stage (Per2:OR=3.47, 95%CI: 1.88∼6.42, *P*<0.001) and further metastasis (Per2:OR=2.35, 95%CI: 1.35∼4.11, *Ρ*=0.003). Furthermore, the results revealed that low expressions of Per1 and Per2 were also correlated with poor overall survival of cancers (Per1: HR=1.35, 95%CI: 1.06∼1.72, *P*=0.014; Per2: HR=1.43, 95%CI: 1.10∼1.85, *P*=0.007). Subgroup analysis indicated that low Per1 and Per2 expressions were especially associated with poor prognosis of gastrointestinal caners (Per1: HR=1.33, 95%CI: 1.14∼1.55, *Ρ*<0.001, *Ι*^2^=4.2%; Per2: HR=1.62, 95%CI: 1.25∼2.18, *P*<0.001, *I*^2^=0.0%).

**Conclusions:**

Our study suggested that low Per1, Per2 and Npas2 expression played a distinct and crucial role in progression of cancers. Low expressions of Per1 and Per2 could serve as unfavorable indicators for cancers prognosis, especially for gastrointestinal cancers.

## Introduction

Period1 (Per1), period2 (Per2), period3 (Per3), cryptochrome1 (Cry1), cryptochrome2 (Cry2), aryl hydrocarbon receptor nuclear translocator-like protein 1 (Bmal1), neuronal PAS domain protein 2 (Npas2) and circadian locomoter output cycles protein kaput (CLOCK) genes are the eight core circadian clock genes that generate and maintain circadian rhythms in many physiologic processes [1, 2]. Per1, Per2 and Per3 were reported to play an important role in regulating cancer cell growth, proliferation and apoptosis [3, 4, 5]. Cry1 and Cry2 acted as transcriptional regulators and checkpoint proteins for cancer cell proliferation and cell cycle control [6, 7]. Bmal1 and Npas2 could regulate cancer cell proliferation and invasion through suppressing the transcription of c-Myc[8, 9]. CLOCK might interact with HIF-1α/ Bmal1 and activate VEGF to stimulate tumor angiogenesis and metastasis [10]. These eight circadian clock genes take part in the carcinogenesis and development of many cancers. Recent studies demonstrated that disrupted expression of these genes was associated with poor progression and prognosis of cancers. For example, low Per1, Per2 and Per3 expressions in different cancers were found to correlate with worse histological grade and poor prognosis [3, 5, 11–22]; Cry2 was reported downregulated in breast and pancreatic cancer and its low expression was associated with higher tumor grade and shorter survival time [23, 24]; reduced Bmal1 and CLOCK expressions were confirmed to result in poor outcome of colon, pancreatic, kidney, head and neck cancers [15, 25, 26]; low Npas2 expression was also found to be related with worse overall survival (OS) in colorectal and breast cancers [9, 27]. However, several other studies showed that low expression of these genes was not correlated with the prognosis of cancers. For example, low Per1 expression in lung cancer was not related to prognosis [28]; downregulated Per2 and Per3 expression were not correlated with gastric and colorectal cancer (CRC) prognosis [29, 30]; low Cry1 expression was not an independent prognostic factor for ovarian cancer [31];reduced expression of Bmal1 and CLOCK were not associated with lung cancer survival and CRC outcomes[28, 30]; Moreover, some studies even implied that overexpression of some of these genes was associated with unfavorable prognosis in patients with cancers. For example, Cry1 and Cry2 overexpression was associated with poor OS in gastric cancer and CRC [29, 32, 33]; Npas2 was frequently upregulated in hepatocellular carcinoma (HCC) and its overexpression significantly contributed to poor prognosis of HCC patients [34]. The clinicopathological and prognostic value of these circadian clock genes in cancers remains controversial and inconclusive. Therefore, we conducted this meta-analysis by integrating published data and online database to clarify the influence of low expression of these seven circadian clock genes on the clinicopathological features and prognosis of different cancers.

## Materials and methods

### 1. Literature search

We systematically searched through the databases (Cochrane Central Register of Controlled Trials, Embase, EBSCO, Ovid, PubMed, Science Direct and Wiley Online Library, China National Knowledge Infrastructure (CNKI) and Wan Fang database) to obtain relevant articles that were published before 1 January 2020. The following terms and phrases were used as search criteria: ‘circadian clock gene’ or ‘period1 (Per1)’ or ‘period2 (Per2)’, or ‘period3 (Per3)’ or ‘cryptochrome1 (Cry1)’ or ‘cryptochrome2 (Cry2)’ or ‘aryl hydrocarbon receptor nuclear translocator-like protein 1 (Bmal1)’ or ‘neuronal PAS domain protein 2 (Npas2) ’ or ‘circadian locomoter output cycles protein kaput (CLOCK)’ and ‘neoplasm’ or ‘tumor’ or ‘cancer’, ‘carcinoma’ and ‘prognosis’ or ‘overall survival (OS)’ or ‘mortality’ or ‘clinic outcome’ or ‘clinicopathological feature’ or ‘odd ratio (OR)’ or ‘hazard ratio (HR)’. The title and abstract of each study obtained in the search was scanned to exclude any clearly irrelevant ones. The remaining articles were reviewed, analyzed, evaluated to determine whether they contained information on the topic of interest. The reference lists of these articles with information on the topic were also reviewed for additional pertinent studies.

### 2. Inclusion and exclusion criteria

Inclusion criteria were as follows: (1) patients diagnosed with cancers; (2) immunohistochemical (IHC) analysis, quantitative PCR, RNA-Sequence analysis and in situ hybridization detection of circadian clock genes expression in tissues; (3) relationships between abnormal expression of circadian clock genes and clinicopathological features or prognostic indicators that were evaluated; (4) odds ratio (OR), hazard ratio (HR) and 95% confidence intervals (CI) that could be obtained directly or indirectly calculated based on the data provided in the graphics and tables; (5) only the newest studies were retained if the data were repeated in different studies and (6) studies in English or Chinese.

Exclusion criteria were as follows: (1) cell or animal studies, letters, case reports, reviews and meta-analyses; (2) articles with similar content or those with small sample sizes (≤ 10) and (3) articles with language barriers.

### 3. Data extraction

The articles that met the criteria were reviewed by two independent investigators (ZhimoWang and HongLv) and extracted data on author, year of publication, nationality, sample size, patient age, detection method, clinical stage and pathological degree. Discrepancies in terms of data extraction were resolved by discussion among all the authors.

### 4. Statistical analysis

The ORs and 95%CI between aberrant circadian clock genes expression and clinicopathological indexes were calculated from the original data in articles using statistical software. The prognostic effects of low circadian clock genes expression were detected by merging the HRs and 95%CI of the included literatures using the forest plot. The HRs and 95% CI values either came from direct extraction of the original text or indirect extraction of survival curve through Engauge Digitizer version 4.1 (https://sourceforge.net/projects/digitizer/) [35].

Heterogeneity was measured by *Q* statistics as follows: no heterogeneity: 0<*Ι*^2^<25%; low heterogeneity: 25%≤*Ι*^2^<50%; moderate heterogeneity: 50%≤*Ι*^2^<75%; high heterogeneity: 75%≤*Ι*^2^≤100%. A random effects model was used to pool HRs and ORs with or without significant heterogeneity. An *I*^2^*<*50% was considered acceptable, and a P value*>*0.10 signified an acceptable degree of homogeneity. Sensitivity and subgroup analysis for the source of the heterogeneity was performed according to publication year, population, detecting method, pathological types and patient number. Publication bias was detected by Begg’s funnel plot and Egger’s test. A two-sided *Ρ* value <0.05 was considered to indicate statistical significance. Statistical analyses were carried out with Stata SE 12.0, Engauge, Microsoft Office 2007.

## Results

### 1. Eligible studies

**Fig 1:**
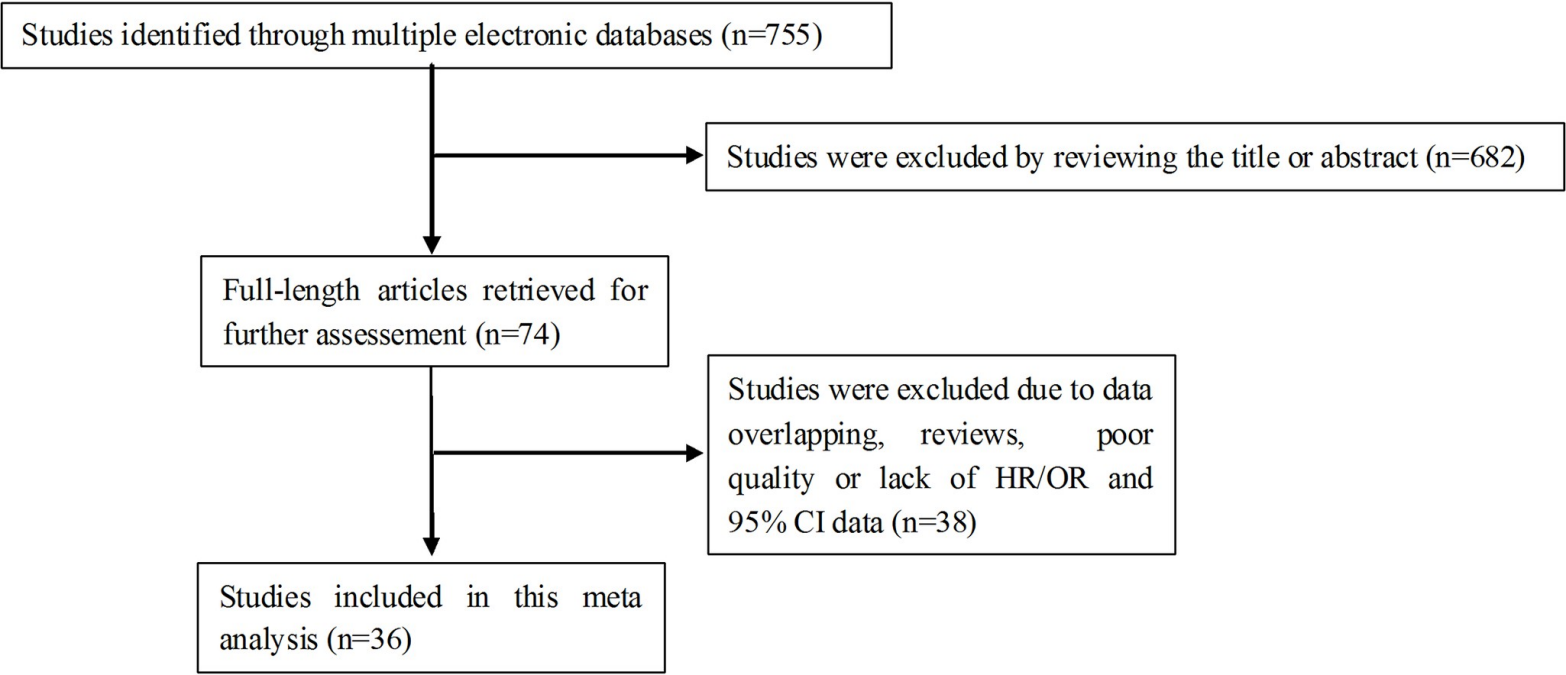
Flow diagram of the study selection process.

**Fig 2:**
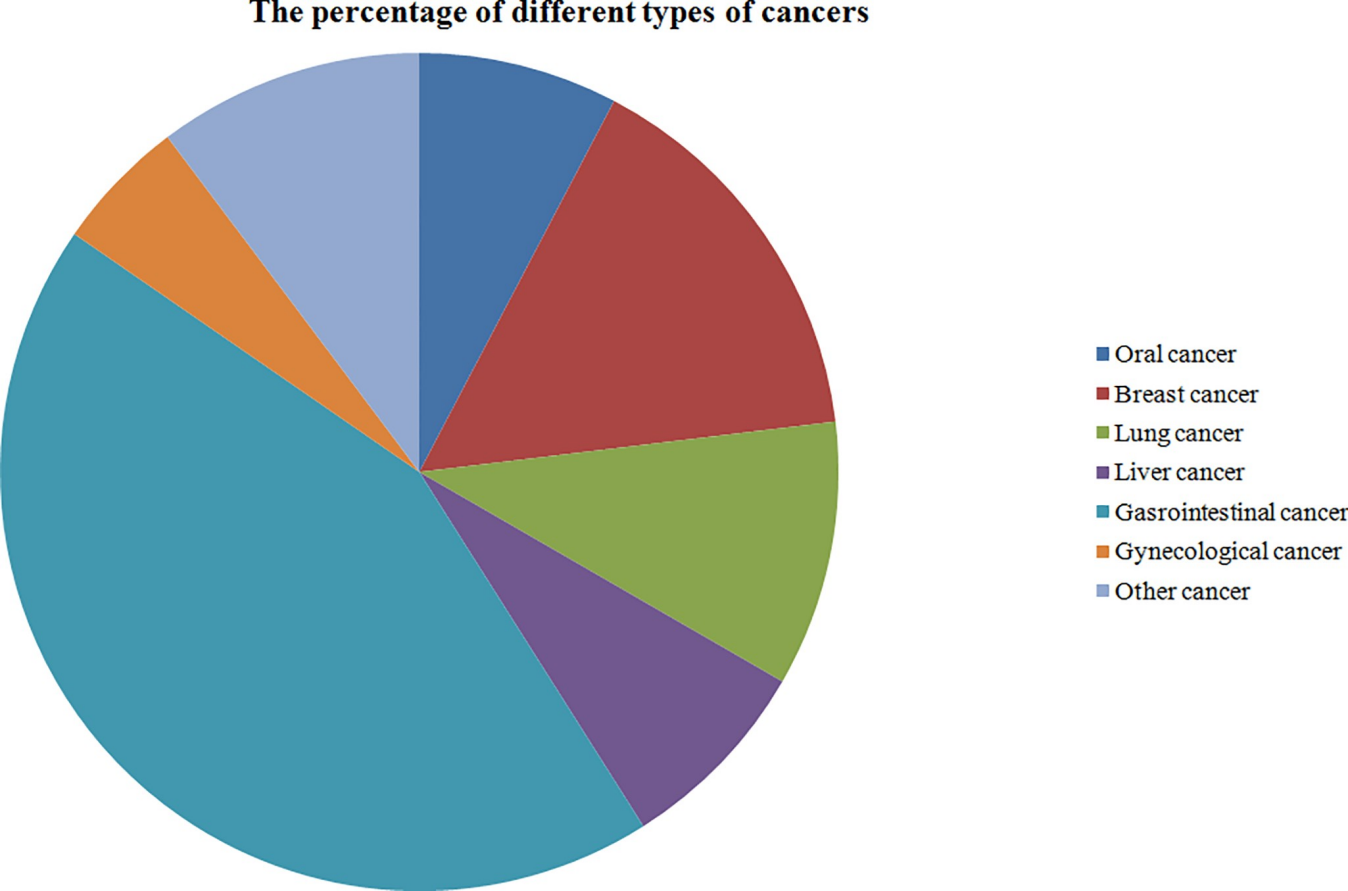
The percentage of different types of cancer included for the meta-analysis.

A total of 755 articles were identified from a search of the included databases using the search strategy as described in Fig 1. 682 articles were excluded through reviewing the titles and abstracts. The remaining 74 articles were then fully examined for their fit with the current meta-analysis, and a further 38 articles were excluded because they met one or more of the exclusion criteria. The final 36 studies [3, 6, 9, 11–17, 19, 22, 24, 25, 27–30, 32–34, 36–50] with 7476 cases were included in our meta-analysis (Fig 1). The fundamental features of the included studies were presented in Table 1 and Fig 2. Among the 36 studies, 25 studies [3, 6, 9, 11–17, 19, 22, 28, 30, 33, 36–41, 43, 46, 47, 50] assessed the association between low circadian clock genes expression and clinicopathological features in patients with cancers, and 22 studies [3, 6, 12–15, 19, 22, 24, 27, 28, 29, 30, 32, 33, 34, 41, 42, 47–50] investigated the relationship between low expression of circadian clock genes and OS in multiple cancers.

**Table 1:** Characteristics of studies included for the meta-analysis.

Author	Year	Population	Cancer type	Number of patients	Gender (Male/Female)	Detection method	Gene (Low expression, high expression)
Winter SL, et al.[36]	2007	Canadian	Breast cancer	34	0/34	Quantitative PCR	Per1 (16, 10)
Kuo SJ, et al. [37]	2009	Chinese	Breast cancer	53	0/53	Immunohistochemistry	Per1 (26, 27)
Climent J, et al.[38]	2010	Chinese	Breast cancer	203	0/203	Quantitative PCR	Per3 (36, 167)
Zhang YB, et al. [11]	2015	Chinese	Breast cancer	60	0/60	Immunohistochemistry	Per1 (19, 41), Per2 (13, 47)
Mao Y, et al. [24]	2015		Breast cancer	737	0/737	Microarray	Cry2
Yi C, et al. [27]	2010	American	Breast cancer	287	0/287	Quantitative PCR	Npas2 (94, 193)
Zhao N, et al. [40]	2013	Chinese	Buccal squamous cell carcinoma	38	16/22	Immunohistochemistry	Per1 (6, 32)
Yang C, et al. [14]	2018	Chinese	Cervical squamous cell carcinoma	239	0/239	IlluminaHiSeq-miRNASeq	Per1 (138, 101)
Eisele L, et al. [45]	2009	German	Chronic lymphocytic leukemia	116	82/34	Quantitative PCR	Cry1 (62, 46)
Wang X, et al. [22]	2012	Chinese	Colon cancer	203	86/117	Immunohistochemistry	Per3 (36, 167)
Wang Y, et al. [41]	2015	Chinese	Colon cancer	203	86/117	Immunohistochemistry	Per1 (21, 182)
			Colon cancer	454	240/214	RNA-Seq analysis	CLOCK (258, 196)
Oshima T, et al. [30]	2011	Japanese	Colorectal cancer	202	110/92	Quantitative PCR	Per1 (101, 101), Per2 (101, 101), Per3 (101, 101), Cry1 (101, 101), Cry2 (101, 101), Baml1 (101, 101), CLOCK(101, 101)
Wu S, et al. [42]	2016	Chinese	Colorectal cancer	214		HiSeq platform	Per1 (82, 132)
Hasakova K, et al. [44]	2018	Slovakian	Colorectal cancer	61	38/23	Quantitative PCR	Per2 (31, 30), Cry1 (31, 30), Cry2 (31, 30)
Yu H, et al. [32]	2013	Chinese	Colorectal cancer	168	89/79	Quantitative PCR	Cry1 (67, 101)
Fang L, et al. [33]	2015	Chinese	Colorectal cancer	289	147/142	Immunohistochemistry	Cry2 (165, 124)
Xue X, et al. [9]	2014	Chinese	Colorectal cancer	108	59/49	Quantitative PCR	Npas2 (54, 54)
Yang SF, et al. [47]	2016	Chinese	Colorectal cancer	120	79/41	Quantitative PCR	Npas2 (97, 23)
Zeng Z, et al. [48]	2014	Chinese	Colorectal cancer	82		Immunohistochemistry	Baml1 (46, 36)
Wang Y, et al. [16]	2011	Chinese	Colorectal carcinoma	38	18/20	Immunohistochemistry	Per2 (24, 14)
Momma T, et al. [13]	2017	Japanese	Colorectal carcinoma	51	32/19	In situ hybridization	Per1 (27, 24), Per2 (25, 26), CLOCK(30, 21)
Liu HJ, et al. [46]	2015	Chinese	Gastrointestinal adenocarcinoma	63	40/23	Immunohistochemistry	Cry1 (37, 26)
Hu ML, et al. [29]	2014	Chinese	Gastric cancer	29	20/9	Quantitative PCR	Per1, Per3
Zhao H, et al. [12]	2014	Chinese	Gastric cancer	246	181/65	Immunohistochemistry	Per1 (143, 103), Per2 (160, 86)
Ding HB, et al. [43]	2018	Chinese	Gastric cancer	106	68/38	Immunohistochemistry	Per1 (4, 102), Cry1 (58, 48)
Yuan P, et al. [34]	2017	Chinese	Hepatocellular carcinoma	217		Quantitative PCR	Npas2 (108, 109)
Li B, et al. [50]	2018	Chinese	Hepatocellular carcinoma	158	143/15	Western blot analysis	CLOCK(79, 79)
Qiu MJ, et al. [15]	2019	Chinese	Kidney cancer	530	344/186	RNA-Seq analysis	Per1 (338, 192), Per2 (323, 204), Per3 (308, 219), Cry2 (302, 229), Npas2 (320,207), CLOCK (283, 247)
			Liver cancer	371	250/121	RNA-Seq analysis	Cry2 (219, 151), Npas2 (230, 141)
Qiu MJ, et al. [28]	2019	Chinese	Lung adenocarcinoma	500	230/270	RNA-Seq analysis	Per1 (367, 133), Per2 (333, 167), Per3 (333, 167), Cry1 (298, 202), Cry2 (333, 167), Npas2 (309, 191), Baml1 (310, 190), CLOCK(324, 176)
			Lung squamous cell carcinoma	494	366/128	RNA-Seq analysis	Per1 (326, 168), Per2 (318, 167), Per3 (318, 176), Cry1 (270, 224), Cry2 (293, 201), Npas2 (292, 202), Baml1 (291, 203), CLOCK(332,162)
De Assis LVM, et al. [49]	2018	American	Melanoma	340		RNA-Seq analysis	Baml1 (170, 170)
Chi C, et al. [17]	2013	Chinese	Non-small cell lung cancer	60	38/22	Immunohistochemistry	Per2 (17, 43)
Liu B, et al. [3]	2014	Chinese	Non-small cell lung cancer	130	75/55	Immunohistochemistry	Per1 (44, 86), Per2 (53, 77), Per3 (48, 82)
Chen R, et al. [39]	2012	Chinese	Oral squamous cell carcinoma	41	24/17	Immunohistochemistry	Per1 (7, 34)
Xiong H, et al. [19]	2018	Chinese	Oral squamous cell carcinoma.	40	25/15	Quantitative PCR	Per2 (24, 16)
Tokunaga H, et al. [31]	2008	Japanese	Ovarian cancer	104	0/104	Quantitative PCR	Cry1
Li W, et al. [25]	2016	Chinese	Pancreatic ductal adenocarcinoma	87	51/36	Immunohistochemistry	Baml1 (61, 26)

### 2. Circadian clock genes expression and clinicopathological features of cancers

**Fig 3:**
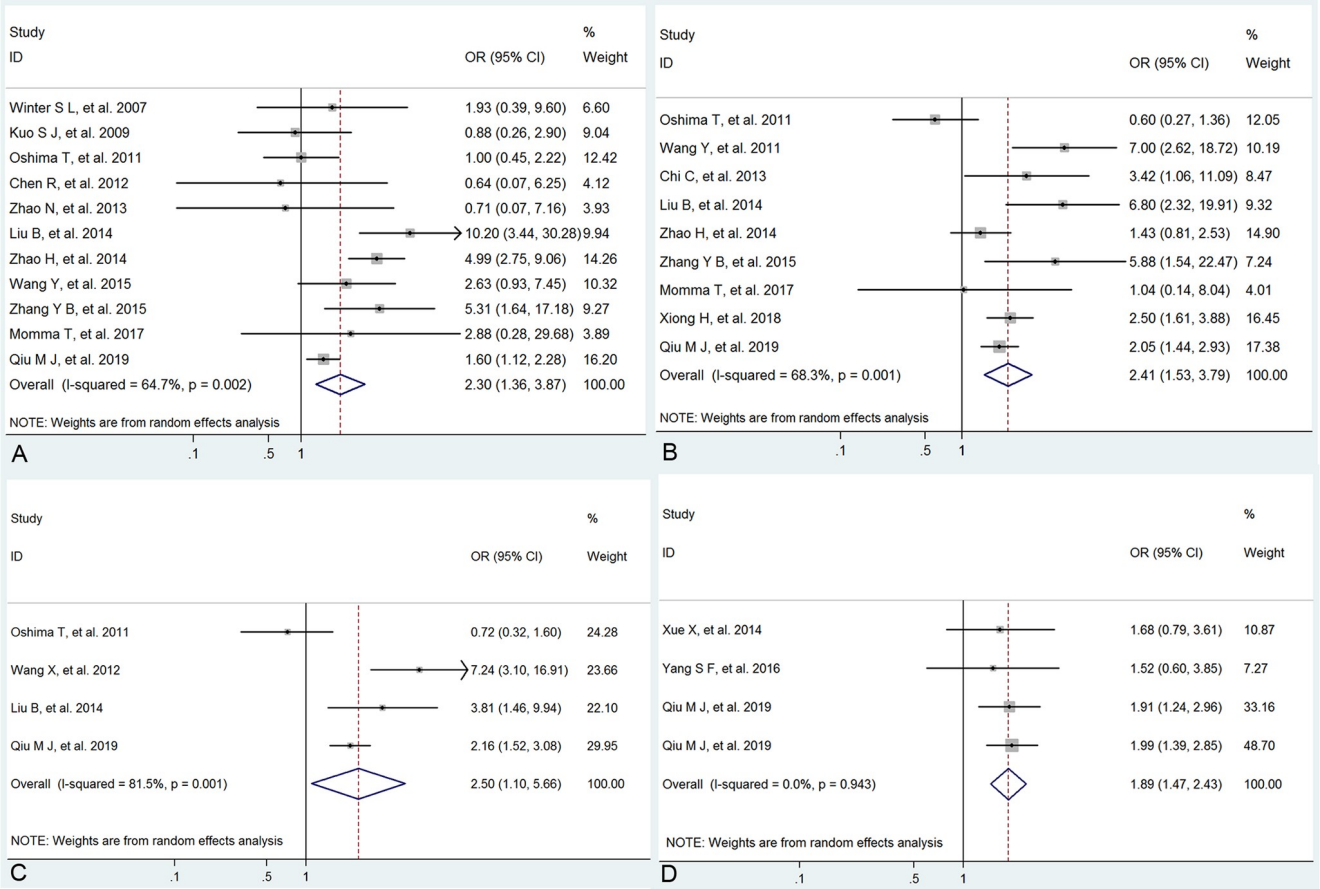
Forrest plot of odds ratio (OR) for the association of low Per1 (A), Per2 (B), Per3 (C) and Npas2 (D) expression and cancer differentiation.

**Fig 4:**
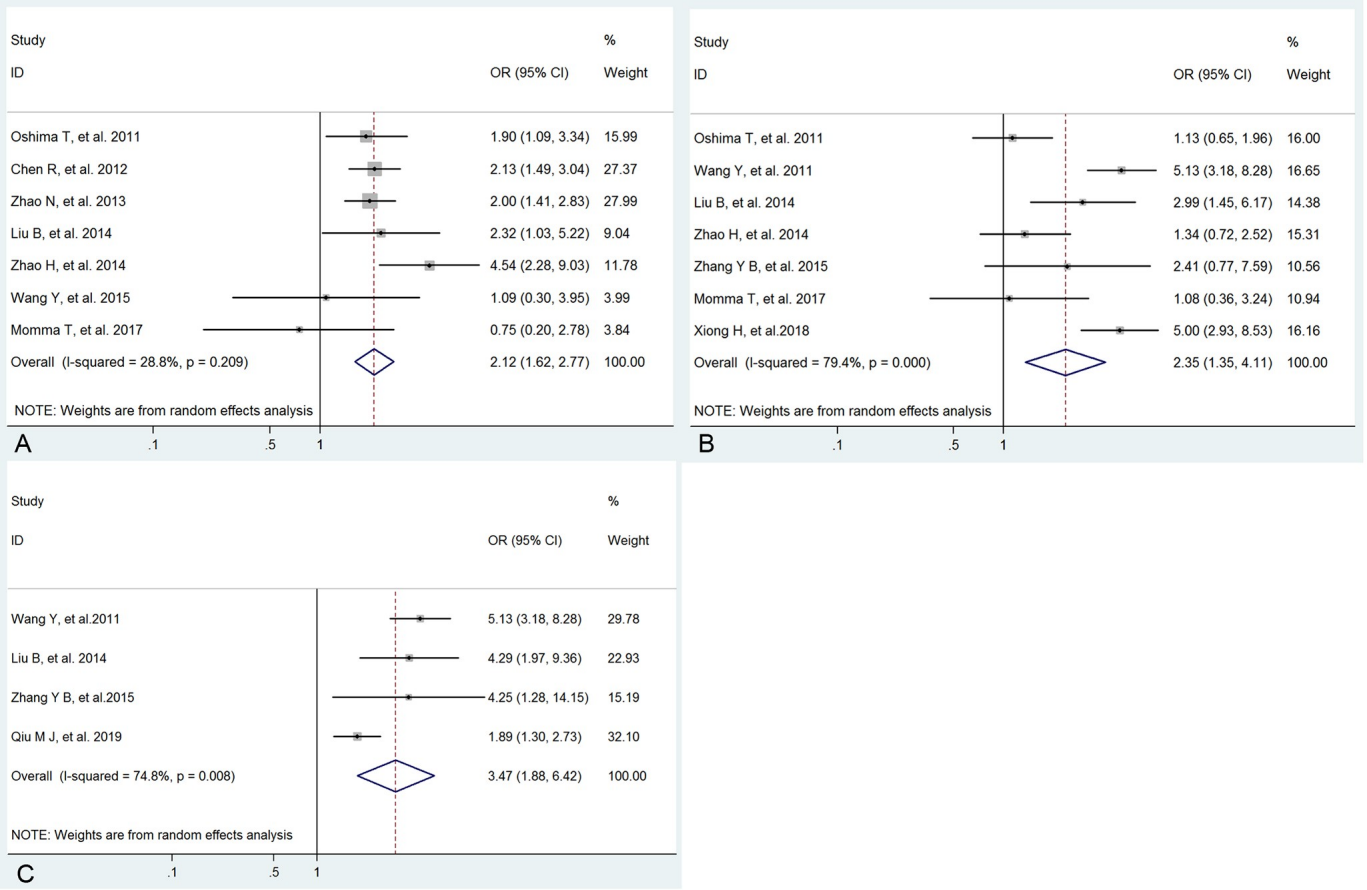
Forrest plot of odds ratio (OR) for the association of low Per1 expression and depth of invasion (A), low Per2 expression and lymph node metastasis (B) and TNM stage (C).

The correlation between low expression of circadian clock genes and clinicopathological features was exhibited in Table 2 and Figs 3 and 4 as follows. The pooled ORs indicated that the low expressions of Per1, Per2, Per3 and Npas2 were significantly related with poor differentiation (Per1: OR=2.30, 95%CI: 1.36∼3.87,=0.002; Per2: OR=2.41, 95%CI: 1.53∼3.79, *Ρ*<0.001; Per3: OR=2.50, 95%CI: 1.10∼5.66, *Ρ*=0.001 and Npas2: OR=1.89, 95%CI: 1.47∼2.43, *Ρ*<0.001), with no heterogeneity to high heterogeneity among studies. Furthermore, we also found that low expression of Per1 was obviously correlated with deeper depth of invasion (OR=2.12, 95%CI: 1.62∼2.77, *Ρ*<0.001; *Ι*^2^=28.8%) and low Per2 expression was significantly associated with more advanced TNM stage (OR=3.47, 95%CI: 1.88∼6.42, *Ρ*<0.001; *Ι*^2^=74.8) and more lymph node metastasis (OR=2.35, 95%CI: 1.35∼4.11, *Ρ*=0.003; *Ι*^2^=79.4). Therefore, although heterogeneity existed, these pooled results suggested that low expressions of Per1, Per2, Per3 and Npas2 might play important roles in the development and progression of cancers.

**Table 2:** Main meta-analysis results of association between low circadian clock genes expression and clinicopathological features in cancers.

Circadian clock gene	Clinicopathological parameters	No. of studies	No. of patients	Pooled OR(95%CI)	Zvalue	P-value	Heterogeneity		Publication bias	
							I^2^ (%)	P-value	Begg’s P value	Egger’s P value
Per1	Differentiation (Moderate+Well/Poor)	11	1588	2.30 (1.36, 3.87)	3.13	0.002	64.7	0.002	1.000	0.832
	Clinical Stage (I+II/III+IV)	7	692	1.85 (0.85, 4.00)	1.56	0.120	84.6	<0.001	0.230	0.168
	Depth of invasion (T1+T2/T3+T4)	7	911	2.12 (1.62, 2.77)	5.49	<0.001	28.8	0.209	0.368	0.601
	Lymph node metastasis (Absent/Present)	9	1051	1.98 (0.77, 5.09)	1.42	0.155	91.0	<0.001	0.251	0.311
	Tumor size (<5CM/≥5CM)	5	639	0.91 (0.60, 1.36)	0.48	0.630	53.0	0.075	0.806	0.301
Per2	Differentiation (Moderate+Well/Poor)	9	1357	2.41 (1.53, 3.79)	3.78	<0.001	68.3	0.001	0.754	0.525
	Clinical Stage (I+II/III+IV)	4	397	3.87 (0.40, 37.23)	1.17	0.241	96.7	<0.001	0.734	0.623
	Depth of invasion (T1+T2/T3+T4)	6	707	1.88 (0.75, 4.74)	1.35	0.178	89.5	<0.001	1.000	0.847
	TNM (I + II/III + IV)	4	758	3.47 (1.88, 6.42)	3.98	<0.001	74.8	0.008	1.000	0.472
	Lymph node metastasis (Absent/Present)	7	767	2.35 (1.35, 4.11)	3.00	0.003	79.4	<0.001	0.548	0.391
	Tumor size (<5CM/≥5CM)	3	358	0.69 (0.33, 1.42)	1.01	0.314	68.6	0.041	0.296	0.150
Per3	Differentiation (Moderate+Well/Poor)	4	1065	2.50 (1.10, 5.66)	2.19	0.029	81.5	0.001	0.734	0.280
	Depth of invasion (T1+T2/T3+T4)	3	535	2.45 (0.78, 7.89)	1.54	0.124	86.1	0.001	0.296	0.204
	Lymph node metastasis (Absent/Present)	3	535	1.50 (0.81, 2.79)	1.29	0.197	61.2	0.076	0.296	0.173
Cry1	Differentiation (Moderate+Well/Poor)	4	539	0.89 (0.47, 1.68)	0.36	0.722	52.8	0.095	0.734	0.580
	Depth of invasion (T1+T2/T3+T4)	4	539	0.86 (0.22, 3.26)	0.22	0.825	85.5	<0.001	0.734	0.319
	Lymph node metastasis (Absent/Present)	4	539	0.55 (0.29, 1.03)	1.87	0.062	62.9	0.044	0.734	0.453
	Tumor size (<5CM/≥5CM)	4	539	1.10 (0.77, 1.56)	0.52	0.603	0.0	0.464	0.734	0.326
Cry2	Differentiation (Moderate+Well/Poor)	4	1392	1.35 (0.84, 2.15)	1.24	0.214	71.6	0.014	0.308	0.201
	Depth of invasion (T1+T2/T3+T4)	2	491	0.85 (0.44, 1.66)	0.47	0.636	58.6	0.120	1.000	
	Lymph node metastasis (Absent/Present)	2	491	1.11 (0.80, 1.52)	0.61	0.543	0.0	0.791	1.000	
Npas2	Differentiation (Moderate+Well/Poor)	4	1129	1.89 (1.47, 2.43)	4.98	<0.001	0.0	0.943	0.089	0.003
	TNM (I + II/III + IV)	5	2123	0.79 (0.40, 1.55)	0.70	0.486	86.6	<0.001	0.221	0.020
CLOCK	Differentiation (Moderate+Well/Poor)	4	941	0.87(0.66, 1.16)	0.95	0.342	0.0	0.453	1.000	0.707
	TNM (I + II/III + IV)	3	1142	1.08(0.59, 2.00)	0.26	0.798	80.8	0.006	0.296	0.531

There was no significant association between low expressions of Cry1, Cry2, CLOCK and clinicopathological parameters. The combined ORs were 0.89 (95%CI: 0.47∼1.68, *Ρ*=0.722) forCry1 and differentiation, 0.86 (95%CI: 0.22∼3.26, *Ρ*=0.825) for Cry1 and invasion depth and 0.55 (95%CI: 0.29∼1.03, *Ρ*=0.062) for Cry1 and lymph node metastasis. The pooled ORs were 1.35 (95%CI: 0.84∼2.15, *Ρ*=0.214) forCry2 and differentiation, 0.85 (95%CI: 0.44∼1.66, *Ρ*=0.636) for Cry2 and invasion depth and 1.10 (95%CI: 0.80∼1.52, *Ρ*=0.543) for Cry2 and lymph node metastasis. The pooled ORs were 0.87 (95%CI: 0.66∼1.16, *Ρ*=0.342) forCLOCK and differentiation, 1.08 (95%CI: (0.59∼2.00, *Ρ*=0.798) forCLOCK and TNM stage.

To explore the heterogeneity among these results, we conducted the subgroup analysis. The results indicated that the correlation between low Per1 expression and differentiation was exhibited in non-IHC group (OR=1.51, 95%CI: 1.10∼2.08, *Ρ*=0.010) and published after 2015 group (OR=1.62, 95%CI: 1.14∼2.30, *Ρ*=0.007) without heterogeneity (*Ι*^2^=0.0%, *Ρ*=0.680 and *Ι*^2^=0.0%, *Ρ*=0.625, respectively). The relationship between low Per2 expression and differentiation was displayed in non-gastrointestinal cancer group (OR=2.82, 95%CI: 1.91∼4.15, *Ρ*<0.001, *Ι*^2^= 37.2%, *Ρ*=0.173) and published before 2015 group (OR=2.19, 95%CI: 1.66∼2.88, *Ρ*<0.001, *Ι*^2^=0.0%, *Ρ*=0.606). The correlation between low Per2 expression and TNM was also exhibited in IHC group (OR =4.82, 95%CI: 3.27∼7.08, *Ρ*<0.001) and published before 2015 group (OR=4.82, 95%CI: 3.27∼7.08, *Ρ*<0.001) without heterogeneity (^2^=0.0%, *Ρ*=0.908 and *Ι*^2^= 0.0%, *Ρ*=0.908, respectively). Furthermore, the heterogeneity among studies related to low Per2 expression and lymph node metastasis was obviously decreased in non-gastrointestinal cancer group (OR=3.89, 95%CI: 2.59∼5.84, *Ρ*<0.001, *Ι*^2^=1.4%, *Ρ*=0.363). Additionally, the heterogeneity among studies related to low Per3 expression and differentiation was also decreased in non-gastrointestinal cancer group (*Ι*^2^= 5.6%, *Ρ*=0.276) and IHC group (*Ι*^2^=0.0%, *Ρ*=0.326) (Table 3). These results indicated that the differences in detecting methods, publish years and pathological types might be the source of study heterogeneity.

**Table 3:** Subgroup analysis results of association between low circadian clock genes expression and clinicopathological and prognostic parameters in cancers.

Circadian clock gene	Clinicopathological parameters		No. of studies	No. of patients	Pooled HR or OR (95%CI)	Z value	P-value	Heterogeneity		Publication bias	
								I^2^ (%)	P-value	Begg’s P value	Egger’s P value
Per1	Overall survival(Low/High)	Chinese	10	2585	1.33 (1.02, 1.74)	2.09	0.037	80.0	**<**0.001	0.592	0.183
		Non-Chinese	2	253	1.52 (1.02, 2.28)	2.05	0.041	8.6	0.296	1.000	
		Gastrointestinal cancer	7	945	1.33 (1.14, 1.55)	3.59	**<**0.001	4.2	0.395	1.000	0.528
		Non-gastrointestinal cancer	5	1893	1.37 (0.86, 2.17)	1.32	0.188	89.2	**<**0.001	0.086	0.054
		IHC	3	579	2.22 (1.29, 3.80)	2.89	0.004	64.4	0.060	1.000	0.509
		Non-IHC	9	2259	1.16 (0.92, 1.46)	1.25	0.21	69.5	0.001	0.754	0.378
		Number of patients ≥100	9	2660	1.38 (1.00, 1.89)	1.98	0.048	82.1	**<**0.001	0.348	0.069
		Number of patients <100	3	178	1.30 (0.97, 1.75)	1.78	0.075	24.0	0.268	1.000	0.784
		Published before 2015	5	162	1.66 (1.15, 2.38)	2.72	0.007	72.3	0.006	0.462	0.202
		Published after 2015	7	2028	1.17 (0.85, 1.60)	0.96	0.335	75.8	**<**0.001	0.764	0.268
	Differentiation (Moderate+Well/Poor)	Chinese	8	1301	2.61 (1.39, 4.92)	2.97	0.003	71.3	0.001	0.711	0.757
		Non-Chinese	3	287	1.23 (0.62, 2.44)	0.60	0.546	0.0	0.585	0.296	0.061
		Gastrointestinal cancer	4	702	2.46 (1.01, 6.04)	1.97	0.049	70.1	0.018	1.000	0.733
		Non-gastrointestinal cancer	7	886	2.16 (1.04, 4.51)	2.06	0.039	62.9	0.013	1.000	0.761
		IHC	7	771	2.95 (1.46, 5.98)	3.01	0.003	59.5	0.022	0.230	0.148
		Non-IHC	4	817	1.51 (1.10, 2.08)	2.58	0.01	0.0	0.680	1.000	0.914
		Number of patients ≥100	5	1311	2.77 (1.34, 5.74)	2.74	0.006	81.7	**<**0.001	0.462	0.412
		Number of patients **<**100	6	277	1.76 (0.83, 3.71)	1.47	0.141	19.5	0.286	1.000	0.473
		Published before 2015	9	1007	2.39 (1.24, 4.59)	2.60	0.009	65.7	0.003	0.466	0.302
		Published after 2015	2	581	1.62 (1.14, 2.30)	2.70	0.007	0.0	0.625	1.000	
Per2	Overall survival(Low/High)	Chinese	6	1940	1.40 (1.04, 1.89)	2.22	0.026	74.5	0.001	0.060	0.088
		Non-Chinese	3	324	1.74 (1.00, 3.03)	1.96	0.05	0.0	0.689	0.296	0.137
		Gastrointestinal cancer	4	570	1.65 (1.25,2.18)	3.50	**<**0.001	0.0	0.851	0.308	0.641
		Non-gastrointestinal cancer	5	1694	1.36 (0.96, 1.93)	1.75	0.08	75.7	0.002	0.221	0.114
		IHC	2	376	1.92 (1.24, 2.96)	2.93	0.003	47.1	0.169	1.000	
		Non-IHC	7	1888	1.24 (0.95, 1.60)	1.59	0.111	49.3	0.066	0.548	0.215
		Number of patients ≥100	6	2102	1.39 (1.05, 1.83)	2.33	0.02	73.0	0.002	0.133	0.078
		Number of patients **<**100	3	162	1.92 (0.84, 4.36)	1.55	0.122	5.1	0.349	0.296	0.023
		Published before 2015	3	578	1.85 (1.42, 2.392)	4.62	**<**0.001	0.0	0.370	1.000	0.367
		Published after 2015	6	1686	1.15 (0.90, 1.47)	1.10	0.27	43.1	0.118	0.707	0.379
	Differentiation (Moderate+Well/Poor)	Chinese	7	1104	2.89 (1.92, 4.35)	5.08	**<**0.001	57.7	0.028	0.368	0.057
		Non-Chinese	2	253	0.65 (0.31, 1.37)	1.14	0.256	0.0	0.621	1.000	
		Gastrointestinal cancer	4	537	1.62 (0.58, 4.54)	0.91	0.363	79.3	0.002	1.000	0.860
		Non-gastrointestinal cancer	5	820	2.82 (1.91, 4.15)	5.24	**<**0.001	37.2	0.173	0.221	0.032
		IHC	5	534	3.92 (1.80, 8.54)	3.45	0.001	68.2	0.014	1.000	0.054
		Non-IHC	4	823	1.58 (0.89, 2.81)	1.55	0.12	69.4	0.020	0.734	0.373
		Number of patients ≥100	4	1108	1.75 (0.87, 3.50)	1.58	0.114	78.7	0.003	1.000	0.963
		Number of patients **<**100	5	249	3.40 (2.02, 5.71)	4.61	**<**0.001	28.2	0.234	1.000	0.595
		Published before 2015	3	621	2.19 (1.66, 2.88)	5.60	**<**0.001	0.0	0.606	1.000	0.601
		Published after 2015	6	736	2.85 (1.23, 6.61)	2.45	0.014	79.4	**<**0.001	0.707	0.148
	TNM (I + II/III + IV)	IHC	3	228	4.82 (3.27, 7.08)	7.98	**<**0.001	0.0	0.908	1.000	0.307
		Published before 2015	3	228	4.82 (3.27, 7.08)	7.98	**<**0.001	0.0	0.908	1.000	0.307
	Lymph node metastasis (Absent/Present)	Chinese	5	514	3.14 (1.83, 5.40)	4.15	**<**0.001	71.0	0.008	0.221	0.398
		Non-Chinese	2	253	1.12 (0.68, 1.83)	0.45	0.653	0.0	0.942	1.000	
		Gastrointestinal cancer	4	537	1.77 (0.76, 4.16)	1.31	0.189	86.1	**<**0.001	1.000	0.456
		Non-gastrointestinal cancer	3	230	3.89 (2.59, 5.84)	6.54	**<**0.001	1.4	0.363	0.296	0.294
		IHC	4	474	2.72 (1.39, 5.35)	2.91	0.004	73.5	0.010	0.735	0.553
		Non-IHC	3	293	1.90 (0.62, 5.80)	1.13	0.258	87.7	**<**0.001	1.000	0.749
		Number of patients ≥100	3	578	1.59 (0.92, 2.77)	1.65	0.1	56.7	0.099	0.296	0.193
		Number of patients **<**100	4	189	3.42 (1.89, 6.19)	4.07	**<**0.001	61.8	0.049	0.308	0.083
		Published before 2015	2	91	2.51 (0.56, 11.19)	1.21	0.227	83.5	0.014	1.000	
		Published after 2015	5	676	2.24 (1.15, 4.34)	2.38	0.017	80.5	**<**0.001	1.000	0.743
Per3	Differentiation (Moderate+Well/Poor)	Chinese	3	863	3.63 (1.67, 7.87)	3.26	0.001	72.3	0.027	1.000	0.312
		Gastrointestinal cancer	2	405	2.27 (0.24, 21.83)	0.71	0.477	93.3	**<**0.001	1.000	
		Non-gastrointestinal cancer	2	660	2.39 (1.56,3.66)	4.01	**<**0.001	15.6	0.276	1.000	
		IHC	2	333	5.46 (2.89, 10.31)	5.24	**<**0.001	0.0	0.326	1.000	
		Non-IHC	2	732	1.33 (0.46, 3.87)	0.52	0.604	83.3	0.014	1.000	

### 3. Impact of circadian clock genes expression on overall survival of cancers

**Fig 5:**
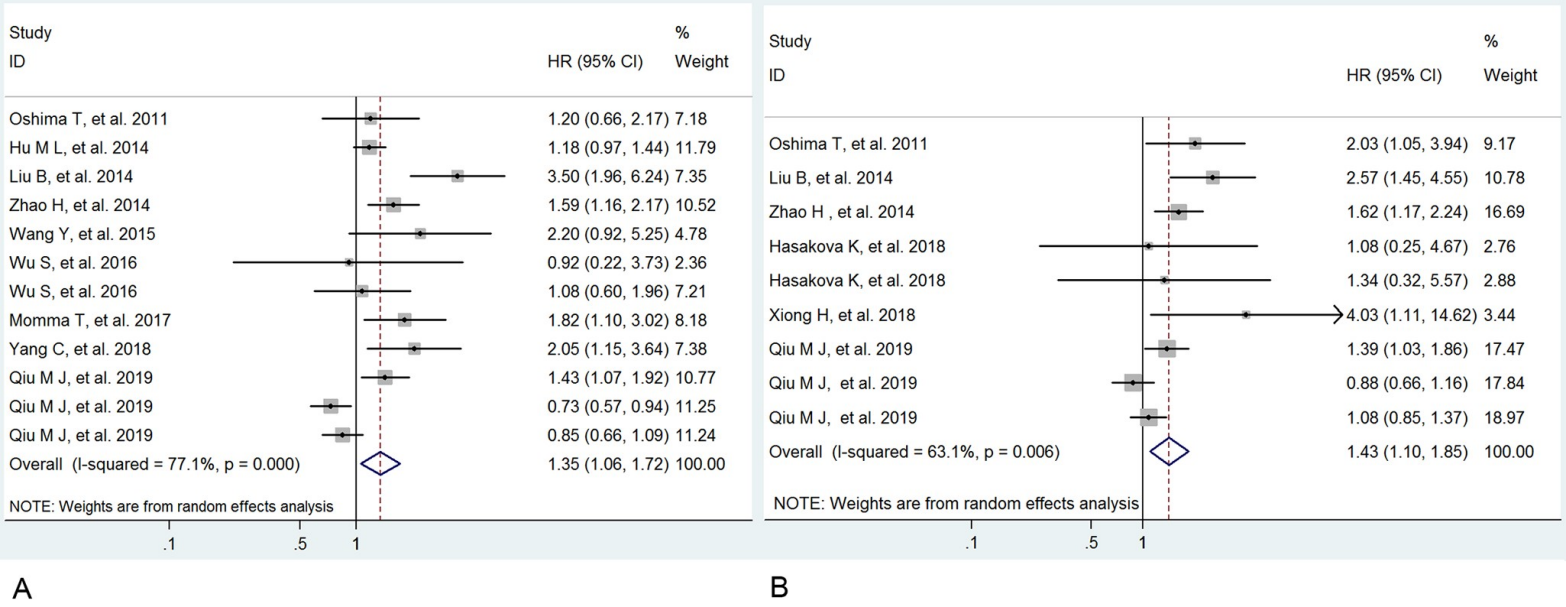
Forrest plot of hazard ratio (HR) for the association of low Per1 (A) and Per2 (B) expression and overall survival.

The association between low expression of circadian clock genes and OS was further explored in this meta-analysis. Low expressions of Per1 and Per2 were related to poor OS in patients with cancers (Per1: HR=1.35, 95%CI: 1.06∼1.72, P=0.014 and Per2: HR =1.43, 95%CI: 1.10∼1.85, P=0.007), with high observed heterogeneity (Per1: *Ι*^2^=77.1%, P<0.001 and Per2: *Ι*^2^=63.1%, P=0.006) (Table 4 and Fig 5). These results demonstrated that low expressions of Per1 and Per2 were significantly associated with worse prognosis in cancers.

**Table 4:** Meta-analysis results of association between low circadian clock genes expression and prognosis in cancers.

Circadian clock gene	No. of studies	No. of patients	Pooled HR(95%CI)	Z	P-value	Heterogeneity		Publication bias	
						I^2^ (%)	P-value	Begg’s P value	Egger’s P value
Per1	12	2838	1.35 (1.06, 1.72)	2.46	0.014	77.1	<0.001	0.537	0.119
Per2	9	2264	1.43 (1.10, 1.85)	2.68	0.007	63.1	0.006	0.602	0.145
Per3	7	2088	1.32 (0.99, 1.76)	1.91	0.056	86.1	<0.001	0.230	0.033
Cry1	8	1706	0.79 (0.54, 1.11)	1.37	0.170	60.8	0.013	0.711	0.849
Cry2	9	3245	1.06 (0.82, 1.37)	0.47	0.635	78.1	<0.001	0.754	0.190
Npas2	7	2519	0.85 (0.61, 1.19)	0.93	0.352	86.0	<0.001	0.548	0.142
Baml1	7	1809	1.10 (0.82, 1.49)	0.64	0.519	75.2	<0.001	0.764	0.438
CLOCK	7	2389	1.05(0.74, 1.48)	0.27	0.790	82.3	<0.001	0.548	0.915

No significant correlation was found between low expression of Per3, Cry1, Cry2, Npas2, Baml1, CLOCK and OS (Per3: HR=1.32, 95%CI: 0.99∼1.76; Cry1: HR=0.79, 95%CI: 0.54∼1.11; Cry2: HR=1.06, 95%CI= 0.82∼1.37; Npas2: HR=0.85, 95%CI: 0.61∼1.19; Baml1: HR=1.10, 95%CI: 0.82∼1.49 and CLOCK: HR=1.05, 95%CI: 0.74∼1.48) (Table 4).

To explain the heterogeneity in OS, subgroup analysis was performed, and the results showed that the heterogeneity among studies related to low Per1 expression and OS was obviously decreased in gastrointestinal cancer group (HR=1.33, 95%CI: 1.14∼1.55, P<0.001, *Ι*^2^=4.2%, P=0.395) and non-Chinese group (HR=1.52, 95%CI: 1.02∼2.08, P=0.041, *Ι*^2^=8.6%, P=0.296) (Table 3). The heterogeneity among studies related to low Per2 expression and OS was significantly decreased in gastrointestinal cancer group (HR=1.62, 95%CI: 1.25∼2.18, P<0.001, *Ι*^2^=0.0%, P=0.851), IHC group (HR=1.92, 95%CI: 1.24∼2.96, P=0.003, *Ι*^2^=47.1%, P=0.169) and published before 2015 group (HR=1.85, 95%CI: 1.42∼2.39, P<0.001, *Ι*^2^=0.0%, P=0.370) (Table 3). The differences in pathological types, populations, detecting methods and publish years might contribute to heterogeneity in these results.

### 4. Sensitivity analysis and publication bias

**Fig 6:**
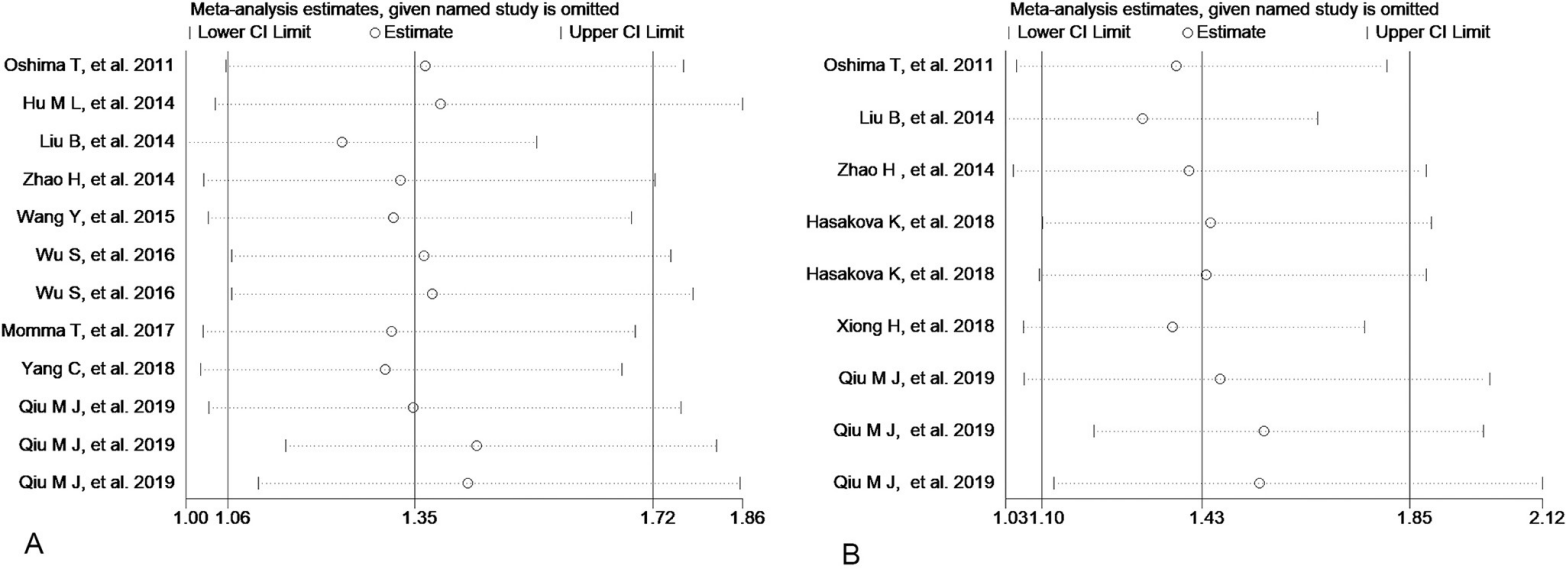
Sensitive analysis of low Per1 (A), Per2 (B) expression and overall survival.

**Fig 7:**
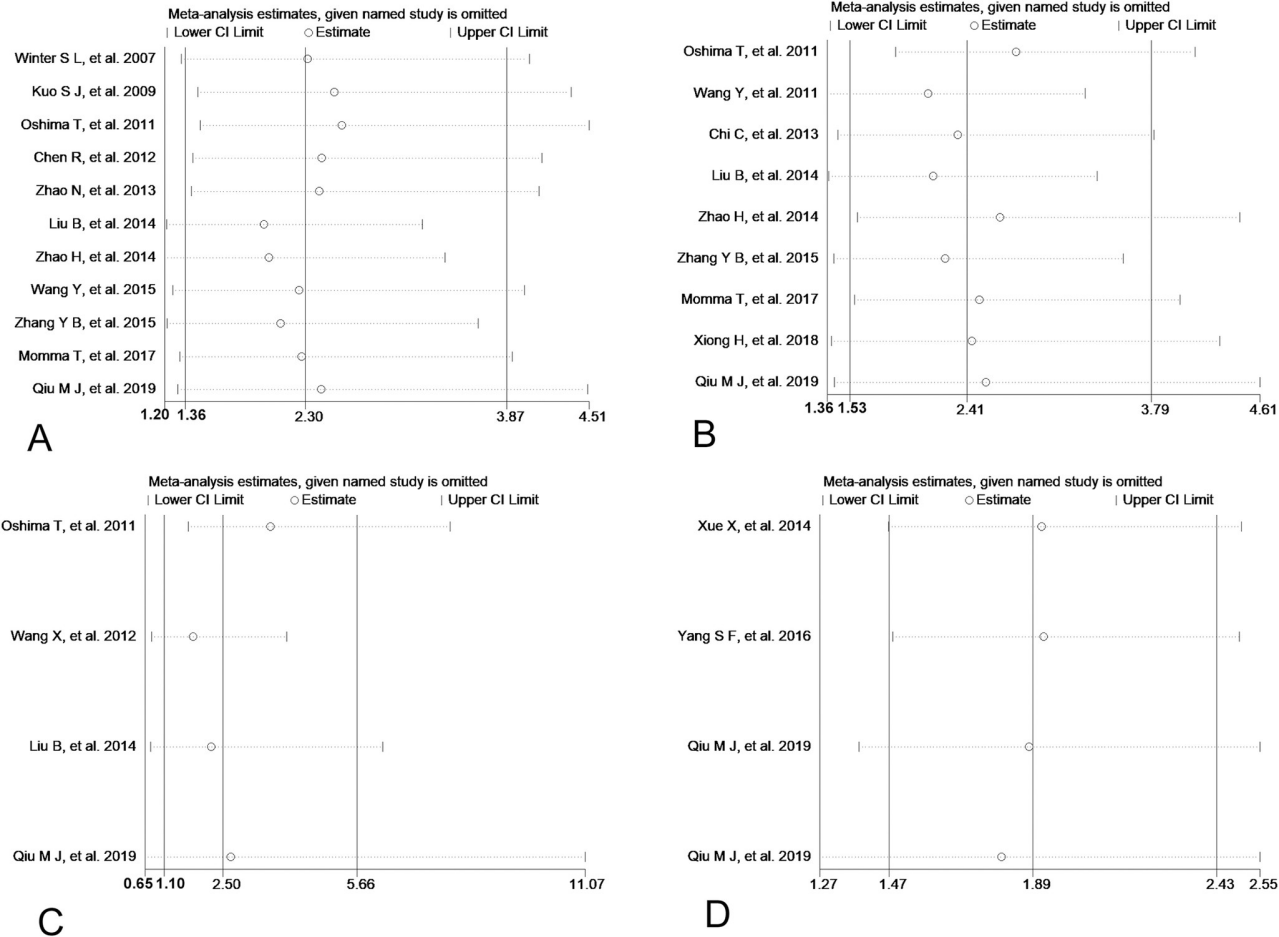
Sensitive analysis of low Per1 (A), Per2 (B), Per3 (C), Npas2 (D) expression and differentiation.

**Fig 8:**
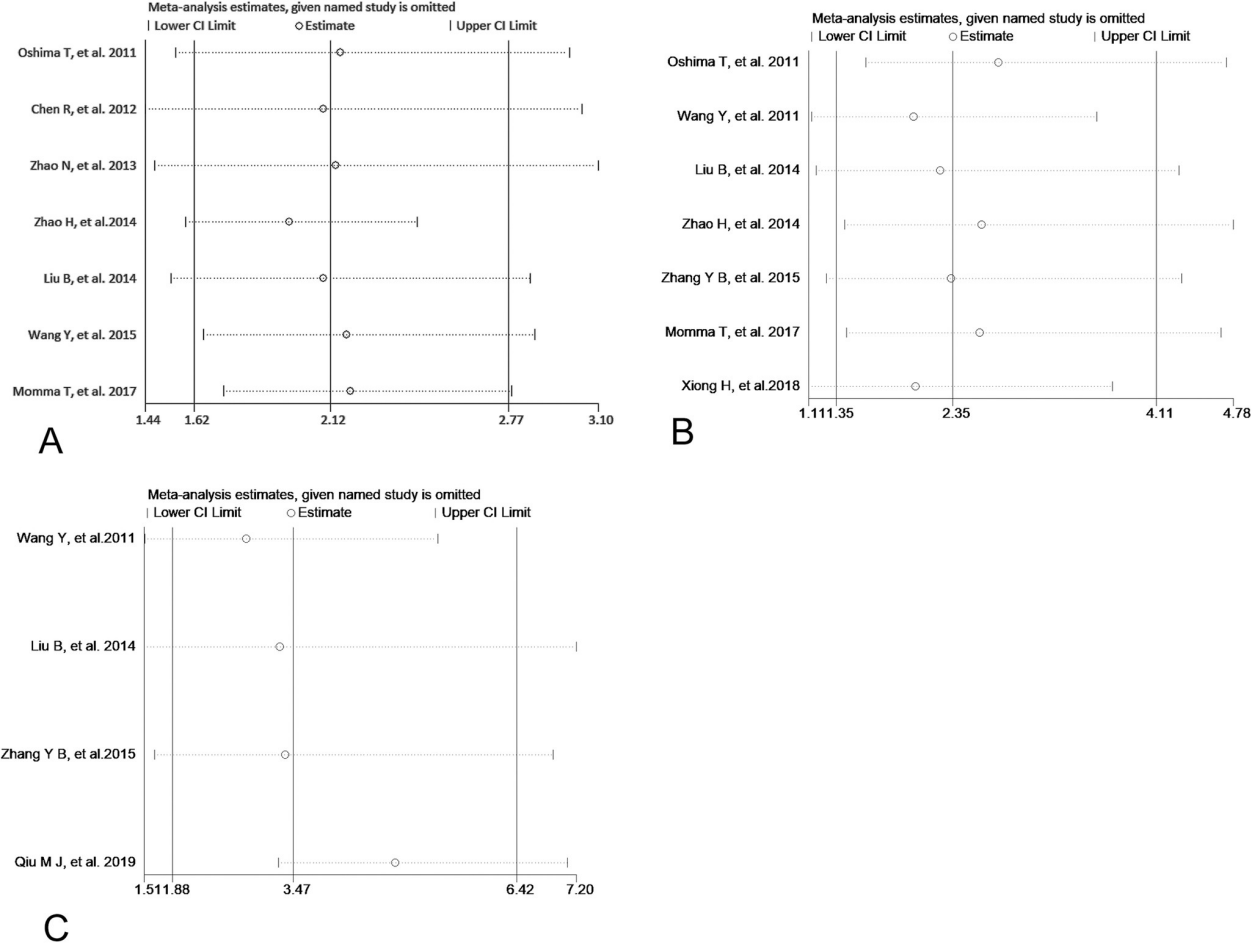
Sensitive analysis of low Per1 expression and depth of invasion (A), low Per2 expression and lymph node metastasis (B),TNM (C).

**Fig 9:**
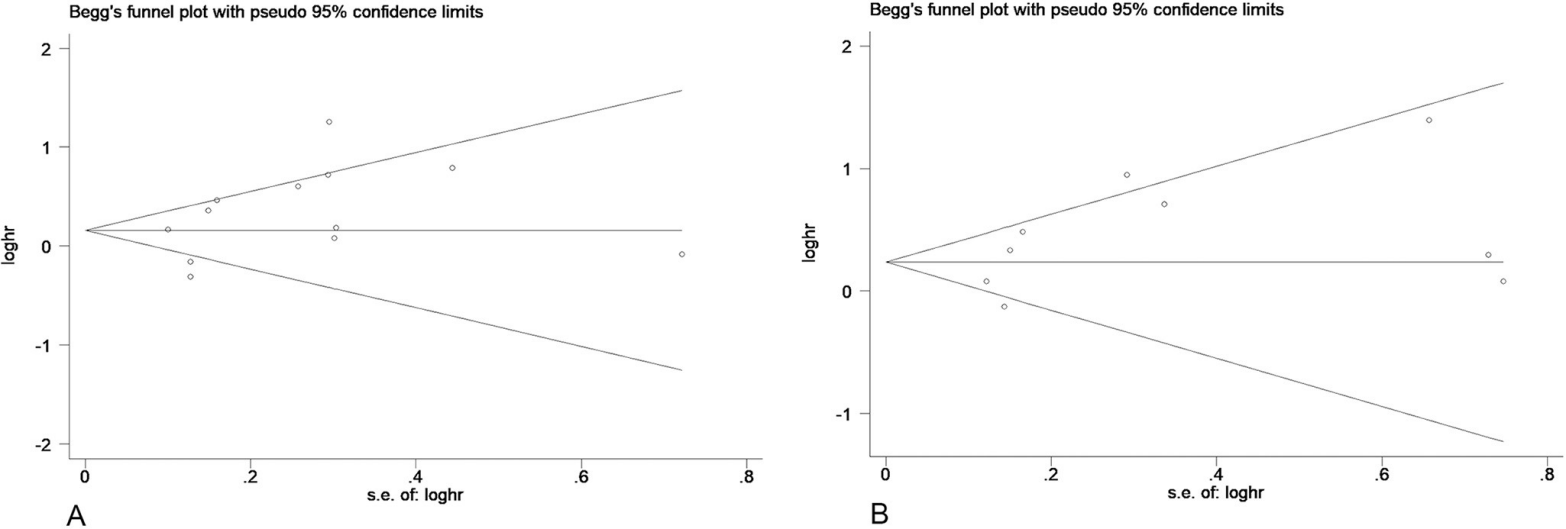
The Begg’s funnel plots assessing the publication bias in analyses of the association of low Per1 (A) and Per2 (B) expression and overall survival.

**Fig 10:**
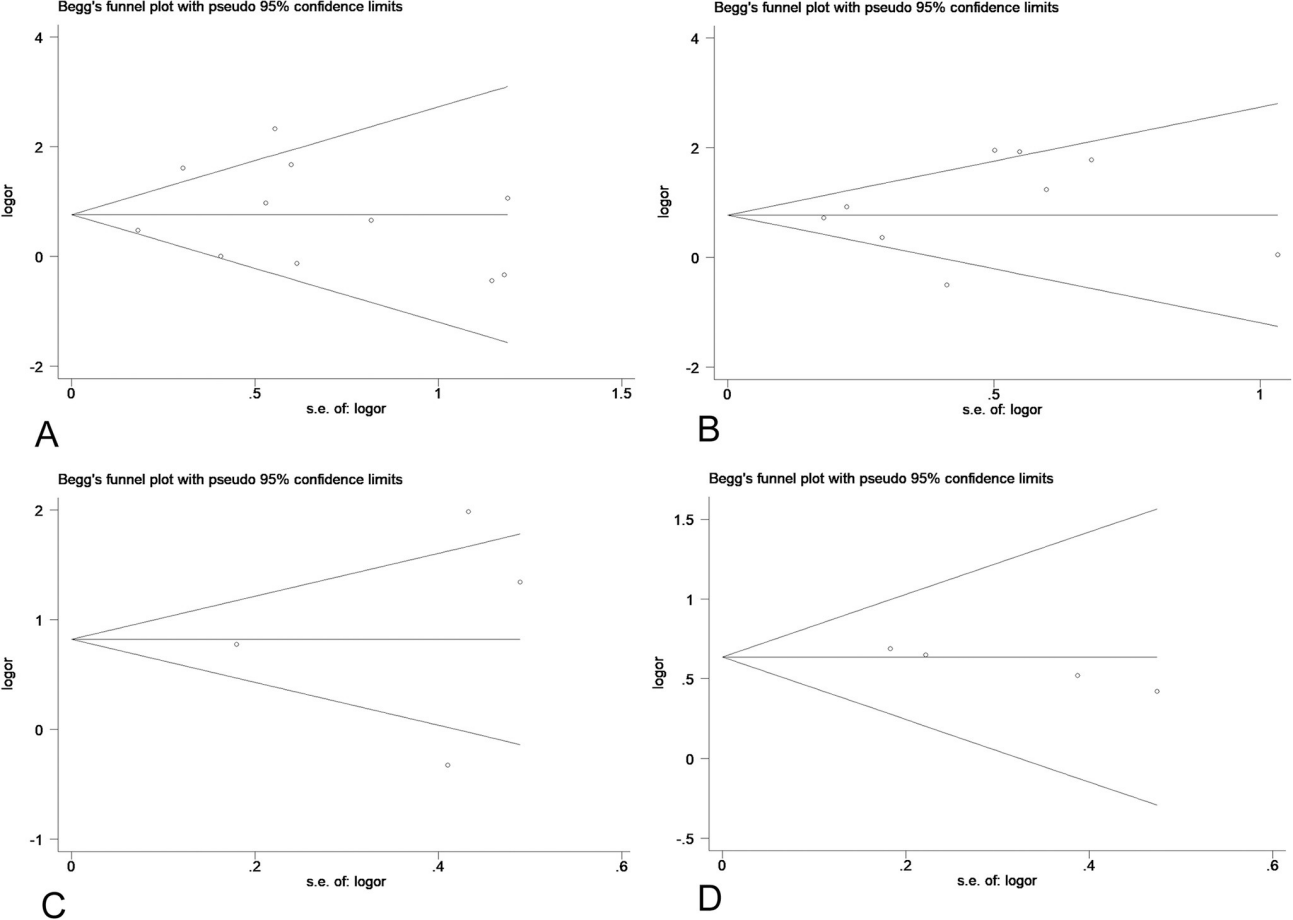
The Begg’s funnel plots assessing the publication bias in analyses of the association of low Per1 (A), Per2 (B), Per3 (C) and Npas2 (D) expression and differentiation.

**Fig 11:**
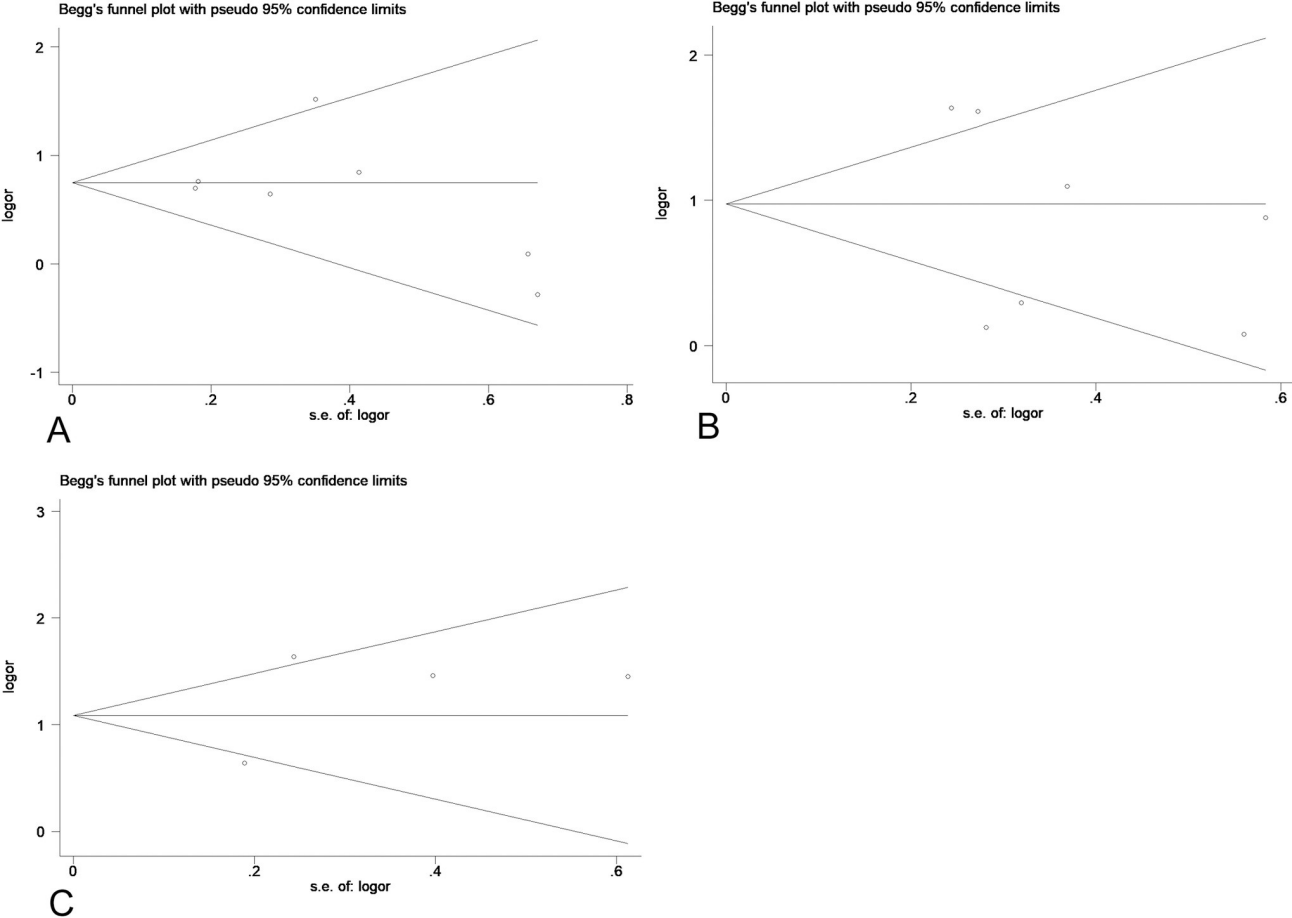
The Begg’s funnel plots assessing the publication bias in analyses of the association of low Per1 expression and depth of invasion (A), low Per2 expression and lymph node metastasis (B), TNM (C).

Sensitivity analysis was performed to check the stability of statistically significant results. As showed in Figs 6, 7 and 8, the pooled ORs or HRs and 95% CIs did not change substantially after removing one study at a time in the comparison between low and high expression of Per1, Per2 and Npas2.However, after removing single study out one by one, the relationship between low expression of Per3 and differentiation become not significant (from OR=2.50, 95%CI: 1.10∼5.66to OR=2.68, 95%CI: 0.65∼11.07) (Fig 7C). These results suggested that the meta-analysis of Per1, Per2 and Npas2 were reliable and stable. Publication bias was detected by Begg’ funnel plot and Egger’s test in the current meta-analysis and the shape of the funnel plots seemed symmetrical in Figs 9, 10 and 11. Thus publication bias might not have a substantial influence on the result of this meta-analysis.

## Discussion

Meta-analysis is a quantitative statistical method that summarizes results of different studies with the same theme to reach a general conclusion. This approach has been successfully used for evaluation of clinicopathological and prognostic parameters in patients with cancers. Circadian clock genes and gene products generate overt circadian rhythms. The disruption of circadian clock genes expression leads to loss of circadian oscillations, such as loss of the 24 h rest-activity cycle, serum corticosterone level daily rhythms, lymphocyte count and body temperature rhythm, which has been associated with higher tumorigenesis rates, faster tumor growth in humans and animal models [42, 51]. However, the relationship between circadian clock genes expression and clinicopathological and prognostic features of cancers was controversial. Therefore, it is rather necessary to analyze and combine these data to reach a reasonable conclusion. Recently, many studies have demonstrated that low expression of circadian clock genes results in the disruption of the normal circadian rhythm and plays an important role in the development, invasion, and metastasis of many kinds of cancers [3, 26, 29, 39], hence, we focused on meta-analyzing the association of low expression of circadian clock genes and cancers. This meta-analysis was the first comprehensive assessment of the association between low circadian clock genes expression and cancer progression and prognosis. Our results showed that low Per1, Per2, Per3 and Npas2 expression played a distinct and crucial role in progression of cancers. Low expressions of Per1 and Per2 could serve as unfavorable indicators for gastrointestinal cancers prognosis.

Inhibition of endogenous Per1 expressionresulted in the abrogation of the ATM/Checkpoint kinase 2 (Chk2) checkpoint pathway and led to less DNA damage-induced apoptosis of cancer cells [52]. Similar effects were also seen when Per2 was knockdown in human leukemia cells. Knockdown of Per2led to downregulation of p53 and upregulation of Cylin B1 and c-Myc and promoted tumorigenesis [53]. Npas2 had been shown to bind to the c-Mycpromoter and suppress its transcription and lower expression of Npas2 resulted in increased cell growth and cycle progression of tumor cells [4, 9]. These observations concurred with our findings and suggested that low expressions of Per1, Per2 and Npas2 could significantly lead to poor differentiation of cancers through the same underlying mechanism mentioned above. Decreased Perl expression upregulated the expression of matrixmetalloproteinase-2 and increased the cell membrane distribution of laminin receptor 1, thereby enhanced tumor cells invasion [54, 55]. This result might partially account for why low Per1 expression was significantly correlated with deeper invasion depth. The epithelial-to-mesenchymal transition (EMT) is a key step in cancer progression and enables cancer cell metastasis. Low expression of Per2 led to the activation of EMT genes TWIST1 and SLUG and promoted cancer metastasis [56]. Therefore, low expression of Per2 might result in further metastasis as our meta-analysis indicated. Since low expressions of Per1 and Per2 were correlated with poorer tumor cell differentiation, deeper invasion depth and worse metastasis, it was reasonable to suppose that low expression of Per1 and Per2 might result in poorer OS, as studies pointed out [3, 11–19]. As expected, the pooled HR results in our study indicated that patients with low expression of Per1 or Per2 had a shorter OS.

Intestinal cell growth, proliferation, differentiation and gut microbiome had a daily rhythm orchestrated by circadian clock genes [57, 58]. Per1 and Per2 were expressed rhythmically throughout the gastrointestinal tract and had been shown to coordinate gastrointestinal functions such as motility, cell proliferation and migration, and regulate host gut microbiota rhythms [59, 60]. The deregulated expression of Per1 and Per2 was correlated with the host and microbiota circadian rhythms disruption and had been thought to be associated with gastrointestinal cancer progression and prognosis [5, 12, 13, 15, 16, 29, 30]. Circadian rhythms controlled by Per1 and Per2 might have stronger and synergistic influence on the gastrointestinal cancer progression, therefore, the heterogeneity among studies which focused on gastrointestinal cancers prognosis was obviously reduced. Further studies are required to investigate the specific mechanisms involved. The heterogeneity among low Per2 expression and differentiation decreased in non-gastrointestinal cancer group (HR=2.82, 95%CI=1.91∼4.15, P<0.001, *I*^*2*^= 37.2%, P=0.173), whereas the heterogeneity of low Per2 expression and OS disappeared in gastrointestinal cancer group (HR=1.62, 95% CI=1.25∼2.18, P<0.001, *I*^*2*^=0.0%, P=0.851). These two results are inconsistent and the reason for this appeared to be that the ORs varied significantly in gastrointestinal cancers and could not reveal the true state since the time variable was not included in the OR analysis. Further studies are needed to illustrate this inconsistency. No association between the low expression of Cry1, Cry2 and Bmal1 and prognosis of cancers was found. The controversial and inconsistent prognostic results in those studies might be the reason for these negative findings [6, 24, 25, 32, 33, 45, 48, 51], and future large cohorts studies are needed to fully evaluate the relationship between the expression of these clock genes and cancer prognosis.

Although some studies focused on other circadian clock genes (such as casein kinase 1ε (CK1ε), receptor subfamily 1 group D member 1/2 (NRD1/2), RAR-related orphan receptor A and B (RORA/B), timeless (Tim) and timeless-interacting protein (Tipin)) and cancers prognosis, these data were not sufficient to meta-analyze HR or OR of these circadian clock genes [13, 23, 29, 49]. The rhythmic expression of clock genes is critical for cancer cell growth, however, only two studies have focused on cosinor analysis of circadian gene expression levels in pancreatic cancer cell lines and tumor bearing mice [40, 61]. Therefore, the rhythmic expression of circadian clock genes was not included in this meta-analysis.

Several limitations do exist in our study. First, potentially relevant unpublished papers and studies published in non-English or Chinese were not included in this meta-analysis, thereby the reliability of our results might be weakened. Second, most of the population in our studies were from Asia, so the conclusion reliability of this meta analysis might also be weakened by this ethic disparity and furtherstudies included more European and American are needed. Third, the sample sizes of the studies ranged from 34 to 737 patients and could be the source of heterogeneity as displayed in Table 4. Fourth, the estimating HRs and their 95% CIs from Kaplan-Meier curves might be less reliable because of the inaccuracy method in extracting survival data. Fifth, we also thought that the difference in published year and detection assays for circadian clock genes expression should be taken into consideration. To the best of our knowledge, immunohistochemistry had been widely used for detecting the expression of circadian clock genes, however, recent researchers preferred performing qRT-PCR or microarray to evaluate circadian clock genes expression. These differences might contribute to the methodological heterogeneity.

## Conclusions

In conclusion, our meta-analysis provided evidence that low Per1, Per2and Npas2 expression played a distinct and crucial role in progression of cancers. Low expressions of Per1 and Per2 could serve as unfavorable indicators for cancers prognosis, especially for gastrointestinal cancers. However, well designed, larger-size and higher-quality cohort studies are needed to investigate the precise impact of Per1, Per2and Npas2 on the pathobiological behaviors and prognosis of cancers.

## Supporting information

S1 Checklist (DOC)

S1 File (DOC)
