# Supplementary material for: Low circadian clock genes expression in cancers: A meta-analysis of its association with clinicopathological features and prognosis
Source: PLoS One. 2020 May 21;15(5):e0233508. doi: 10.1371/journal.pone.0233508 (PMC7241715; doi:10.1371/journal.pone.0233508)
Supplement: S1 File — (DOC) [file pone.0233508.s002.doc]

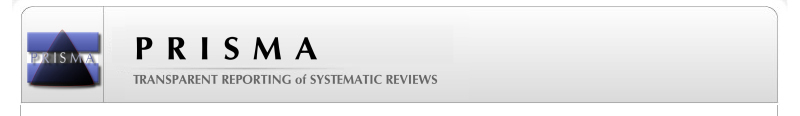
**PRISMA 2009 Flow Diagram**

**Screening**

**Included**

**Eligibility**

**Identification**

Records identified through database searching
(n = 754 )

Additional records identified through other sources
(n = 0 )

Records after duplicates removed
(n = 73 )

Records screened
(n = 73 )

Records excluded
(n = 0 )

Full-text articles assessed for eligibility
(n = 73 )

Full-text articles excluded, with reasons
(n = 38)

Studies included in qualitative synthesis
(n = 35 )

Studies included in quantitative synthesis (meta-analysis)
(n = 35 )
